# First-line Avelumab plus Chemotherapy in Patients with Advanced Solid Tumors: Results from the Phase Ib/II JAVELIN Chemotherapy Medley Study

**DOI:** 10.1158/2767-9764.CRC-23-0459

**Published:** 2024-06-28

**Authors:** Duncan A. Wheatley, Rossana Berardi, Miguel A. Climent Duran, Anna Tomiak, Alastair P. Greystoke, Anthony M. Joshua, Hendrik-Tobias Arkenau, Lajos Géczi, Javier Garciá Corbacho, Luis G. Paz-Ares, Syed A. Hussain, Lubos Petruželka, Angelo Delmonte, Colombe Chappey, Joanna C. Masters, Elisabete Michelon, Danielle A. Murphy, Sandrine Mwewa, Rossano Cesari, Bernard Doger de Spéville

**Affiliations:** 1 Royal Cornwall Hospital, Treliske, Truro, United Kingdom.; 2 AOU delle Marche, Università Politecnica delle Marche, Ancona, Italy.; 3 Instituto Valenciano de Oncología, Valencia, Spain.; 4 Kingston Health Sciences Centre, Kingston, Ontario, Canada.; 5 NU Cancer, Newcastle University, Newcastle upon Tyne, United Kingdom.; 6 St Vincent's Hospital Sydney, Darlinghurst, New South Wales, Australia.; 7 Sarah Cannon Research Institute, HCA Healthcare, London, United Kingdom.; 8 National Institute of Oncology, Budapest, Hungary.; 9 Clinic Institute of Hematological and Oncological Diseases, Hospital Clinic, Barcelona, Spain.; 10 Hospital Universitario 12 de Octubre, Madrid, Spain.; 11 Weston Park Hospital, University of Sheffield, Sheffield, United Kingdom.; 12 General University Hospital in Prague, Prague, Czech Republic.; 13 IRCCS Istituto Romagnolo per lo Studio dei Tumori (IRST) “Dino Amadori,” Meldola, Italy.; 14 Pfizer, San Francisco, California.; 15 Pfizer, San Diego, California.; 16 Pfizer, New York, New York.; 17 Pfizer, Paris, France.; 18 Pfizer, Milan, Italy.; 19 Fundación Jiménez Díaz University Hospital, Madrid, Spain.

## Abstract

**Purpose:**

Chemotherapy can potentially enhance the activity of immune checkpoint inhibitors by promoting immune priming. The phase Ib/II JAVELIN Chemotherapy Medley trial (NCT03317496) evaluated first-line avelumab + concurrent chemotherapy in patients with advanced urothelial carcinoma or non–small cell lung cancer (NSCLC).

**Materials and Methods:**

Avelumab 800 or 1,200 mg was administered continuously every 3 weeks with standard doses of cisplatin + gemcitabine in patients with urothelial carcinoma, or carboplatin + pemetrexed in patients with nonsquamous NSCLC. Dual primary endpoints were dose-limiting toxicity (DLT; phase Ib) and confirmed objective response (phase Ib/II).

**Results:**

In phase Ib, urothelial carcinoma and NSCLC cohorts received avelumab 800 mg (*n* = 13 and *n* = 6, respectively) or 1,200 mg (*n* = 6 each) + chemotherapy. In evaluable patients with urothelial carcinoma treated with avelumab 800 or 1,200 mg + chemotherapy, DLT occurred in 1/12 (8.3%) and 1/6 (16.7%), respectively; no DLT occurred in the NSCLC cohort. In phase II, 35 additional patients with urothelial carcinoma received avelumab 1,200 mg + chemotherapy. Across all treated patients, safety profiles were similar irrespective of avelumab dose. Objective response rates (95% confidence internal) with avelumab 800 or 1,200 mg + chemotherapy, respectively, across phase Ib/II, were 53.8% (25.1–80.8) and 39.0% (24.2–55.5) in urothelial carcinoma, and 50.0% (11.8–88.2) and 33.3% (4.3–77.7) in NSCLC.

**Conclusions:**

Preliminary efficacy and safety findings with avelumab + chemotherapy in urothelial carcinoma and NSCLC were consistent with previous studies of similar combination regimens. Conclusions about clinical activity are limited by small patient numbers.

**Significance:**

This phase Ib/II trial evaluated avelumab (immune checkpoint inhibitor) administered concurrently with standard first-line chemotherapy in patients with advanced urothelial carcinoma or advanced nonsquamous NSCLC without actionable mutations. Efficacy and safety appeared consistent with previous studies of similar combinations, although patient numbers were small.

## Introduction

Avelumab, an anti–programmed cell death ligand-1 (PD-L1) immune checkpoint inhibitor (ICI), is approved in various countries as monotherapy for first-line (1 L) maintenance treatment or second-line treatment of locally advanced or metastatic urothelial carcinoma, monotherapy for metastatic Merkel cell carcinoma, and in combination with axitinib for 1 L treatment of advanced renal cell carcinoma (1, 2). In the phase Ib JAVELIN Solid Tumor trial, 1 L treatment with avelumab showed preliminary antitumor activity and a tolerable safety profile in a cohort of patients with advanced non–small cell lung cancer (NSCLC; ref. 3). In patients with NSCLC and high-expression PD-L1–positive tumors (≥80% of tumor cells) from the phase III JAVELIN Lung 100 trial, numerically improved median overall survival (OS) and progression-free survival (PFS) were observed with 1 L avelumab every 2 weeks or avelumab once weekly versus platinum-based doublet chemotherapy, but results were not statistically significant (4).

Platinum-based chemotherapy is an established treatment for patients with advanced urothelial carcinoma or NSCLC (5, 6). Chemotherapy can have immunostimulatory effects in the tumor microenvironment, including enhancement of antigen presentation, immune cell infiltration, and immunogenicity, providing a mechanism for potential increased antitumor activity when administered in combination with ICIs (7–10). In particular, preclinical and clinical studies suggest that platinum-based chemotherapy can promote immune priming by stimulating MHC class I and increasing immune cell infiltration, and may also increase PD-L1 expression (7, 8). In NSCLC and some other tumors types (e.g., gastric cancer, squamous cell carcinoma of the head and neck, cervical cancer, and triple-negative breast cancer), platinum-based chemotherapy in combination with an ICI is an established treatment approach (11–13). However, in patients with advanced urothelial carcinoma, several phase III trials of ICIs in combination with 1 L platinum-based chemotherapy (or as 1 L monotherapy) did not result in improved OS compared with platinum-based chemotherapy alone (14–16). In contrast, in the phase III JAVELIN Bladder 100 trial, avelumab 1 L maintenance added to best supportive care in patients with advanced urothelial carcinoma that had not progressed with 1 L platinum-based chemotherapy significantly prolonged OS versus best supportive care alone (17, 18). Consequently, avelumab 1 L maintenance has become a standard of care in this setting (6, 19, 20). However, limited data are available for avelumab treatment administered in combination with standard chemotherapy.

Avelumab was initially approved with a weight-based dose of 10 mg/kg every 2 weeks (17, 21–24), but subsequent pharmacokinetic modeling and simulation showed comparable exposure with 800 mg every 2 weeks flat dosing versus weight-based dosing (historical control), leading to the approval of the 800-mg flat dose (1, 2, 25). The optimal dose and schedule for avelumab in combination with chemotherapy has not been defined. Administration of avelumab 800 mg every 3 weeks was predicted to achieve >90% target occupancy [the level of target occupancy associated with clinical activity (26, 27)], and avelumab 1,200 mg every 3 weeks predicted to achieve >90% target occupancy with a similar average serum concentration to the approved 800 mg every 2 weeks dosing regimen. Hence, both regimens could extend the avelumab dosing interval to align with every 3 weeks chemotherapy regimens.

Here we report results from the phase Ib/II JAVELIN Chemotherapy Medley trial (NCT03317496), which evaluated the safety, efficacy, and pharmacokinetics of two flat doses of avelumab (800 or 1,200 mg every 3 weeks) in combination with standard-of-care chemotherapy in cisplatin-eligible patients with advanced urothelial carcinoma or patients with advanced nonsquamous NSCLC. The trial was designed to include an initial phase Ib lead-in to assess safety followed by phase II expansion cohorts.

## Materials and Methods

### Study Design and Participants

JAVELIN Chemotherapy Medley (NCT03317496) was a phase Ib/II, multicenter, open-label trial that investigated avelumab 800 or 1,200 mg every 3 weeks in combination with standard doses and cycles of cisplatin + gemcitabine in patients with urothelial carcinoma or carboplatin + pemetrexed in patients with NSCLC. The study was initially designed to investigate avelumab 800 mg every 3 weeks in combination with chemotherapy, but the protocol was amended to add a 1,200 mg every 3 weeks cohort based on the FDA's recommendation to evaluate an additional dose. The phase Ib lead-in assessed the safety of avelumab 800 or 1,200 mg every 3 weeks in combination with either cisplatin + gemcitabine in patients with urothelial carcinoma or carboplatin + pemetrexed in patients with NSCLC. Enrollment of expansion cohorts in phase II was permitted at the highest dose of avelumab for which the number of patients with dose-limiting toxicity (DLT) in phase Ib was ≤1 of 6 or ≤3 of 12. Although both the urothelial carcinoma and NSCLC cohorts met the criteria for expansion at the highest dose level in phase II, no additional patients were enrolled in the NSCLC cohort per the sponsor's strategic decision (not based on safety concerns). Therefore, phase II assessed preliminary efficacy and further evaluated safety in patients with urothelial carcinoma only, who received avelumab 1,200 mg (the highest dose level of avelumab deemed safe in phase Ib) in combination with cisplatin + gemcitabine. A total of approximately 40 patients with urothelial carcinoma treated at the highest selected dose of avelumab plus chemotherapy, including patients from phase Ib and phase II, would permit objective response rate (ORR) estimation with a maximum SE of 0.079. Patients still on treatment at study termination were enrolled in a continuation study to receive further treatment.

Eligibility criteria included: age ≥18 years; histologically confirmed unresectable locally advanced or metastatic urothelial carcinoma or NSCLC; ≥1 measurable lesion per RECIST version 1.1; Eastern Cooperative Oncology Group performance status of 0 or 1; no prior ICI treatment; no prior systemic treatment for unresectable locally advanced or metastatic disease; and a disease-free interval in patients who had received prior systemic chemotherapy in the adjuvant or neoadjuvant setting (with or without radiotherapy) of ≥6 months for patients with NSCLC and ≥12 months for patients with urothelial carcinoma. Patients with urothelial carcinoma were required to be cisplatin eligible. Patients with NSCLC were required to have a tumor with nonsquamous histology that was wild type for *EGFR*/*ALK*/*ROS1*; patients for whom pembrolizumab monotherapy was available as a standard treatment option were also required to have a tumor proportion score of <50% for PD-L1, as determined by the 22C3 pharmDx or Ventana SP263 PD-L1 IHC assay.

Additional inclusion criteria included: life expectancy of ≥3 months; adequate hepatic function [total bilirubin level of ≤1.5 × the upper limit of normal (ULN), aspartate aminotransferase level of ≤2.5 × ULN, and alanine aminotransferase level of ≤2.5 × ULN]; adequate renal function (estimated creatinine clearance of ≥50 mL/minute); adequate bone marrow function [absolute neutrophil count of ≥1.5 × 10^9^/L; platelet count of ≥100 × 10^9^/L; and hemoglobin level of ≥9 g/dL (transfusion permitted)].

Exclusion criteria included: persistent grade >1 toxicity from prior anticancer therapy per NCI Common Terminology Criteria for Adverse Events (NCI-CTCAE) v4.03; prior grade ≥3 hypersensitivity (NCI-CTCAE v4.03) to platinum-related compounds (all patients), pemetrexed (NSCLC cohort), or gemcitabine (urothelial carcinoma cohort); symptomatic central nervous system metastases requiring steroids; diagnosis of other malignancy ≤2 years prior to enrollment (except adequately treated basal cell or squamous cell skin cancer; carcinoma *in situ* of the bladder, breast, or cervix; or low-grade prostate cancer with no plans for treatment intervention); major surgery or radiotherapy ≤28 days or ≤14 days prior to enrollment, respectively; immunosuppressive agents (except inhaled or topical steroids, local steroid injection, systemic corticosteroids at physiologic doses, or steroids as premedication for hypersensitivity reactions); active infection requiring systemic therapy; and active or history of autoimmune disease that may deteriorate with an immune-stimulatory agent.

This trial was conducted in accordance with the ethical principles of the Declaration of Helsinki and the Good Clinical Practice guidelines, defined by the International Council for Harmonization. All participating patients provided written informed consent. The protocol was approved by the Institutional Review Board or independent ethics committee at each participating center. Investigations were performed in accordance with an assurance filed with and approved by the U.S. Department of Health and Human Services.

### Procedures

Avelumab was administered at a dose of 800 or 1,200 mg as a 1-hour intravenous infusion on day 1 of each 3-week cycle; patients received an antihistamine and acetaminophen prior to the first four infusions. Premedication for subsequent doses was based on clinical judgment and the presence and severity of prior infusion-related reactions. Chemotherapy was administered according to established prescribing information (28–31).

Premedication for carboplatin, cisplatin, and gemcitabine followed local guidelines. Premedication for pemetrexed included folic acid, vitamin B_12_, and dexamethasone according to the U.S. prescribing information or local guidelines. Patients with urothelial carcinoma received chemotherapy until optimal response was achieved. Patients with NSCLC received carboplatin and pemetrexed for a maximum of four to six cycles with pemetrexed maintenance administered at the discretion of the investigator.

Patients received study treatment until disease progression, unacceptable toxicity, patient withdrawal, or study termination by the sponsor. Patients with disease progression who had ongoing clinical benefit based on the investigator's judgment were permitted to continue treatment.

### Endpoints and Assessments

Dual primary endpoints were DLT within the first two treatment cycles (21-day cycles) in phase Ib and confirmed objective response [best overall response of complete response (CR) or partial response (PR)] per RECIST 1.1 by investigator assessment in phase Ib and II. Secondary endpoints included safety [adverse events (AE)/laboratory abnormalities]; duration of response [DOR; assessed from first documentation of CR or PR until progressive disease, death, or last tumor assessment]; time to tumor response (TTR), and PFS per RECIST 1.1; OS; pharmacokinetics; biomarker analyses; and immunogenicity.

DLT was defined as the occurrence of any of the following within the DLT observation period (first two cycles of treatment in the phase Ib lead-in): (i) hematologic AEs, including grade 4 neutropenia lasting for >7 days, febrile neutropenia, neutropenic infection, grade ≥3 thrombocytopenia with bleeding, grade 4 thrombocytopenia, and grade 4 anemia; (ii) nonhematologic AEs, including grade 4 toxicities, grade 3 toxicities lasting for >3 days despite adequate medical management (except endocrinopathies controlled with hormonal treatment), potential Hy law cases, and persistent grade 3 QT interval corrected using Fridericia formula prolongation; and (iii) ≥3-week delay in scheduled administration due to persisting treatment-related toxicities or failure to deliver ≥75% of the planned doses during the first two cycles of treatment due to treatment-related toxicities.

AEs were classified and graded according to the NCI-CTCAE v4.03. Antitumor activity was assessed radiologically every 6 weeks for the first year followed by every 12 weeks thereafter.

### Biomarker Analyses

Biomarker analyses were performed on baseline tumor tissue (archived tissue or fresh biopsy). PD-L1 expression was determined using the Ventana PD-L1 SP263 IHC assay. In the urothelial carcinoma cohorts, PD-L1–positive status was defined using an algorithm that combines assessments of PD-L1 staining on tumor and immune cells, which were scored by pathologists (32); in the NSCLC cohorts, PD-L1–positive status was defined as PD-L1 expression on ≥1% of tumor cells. Whole-exome and whole-transcriptome sequencing was performed on baseline tumor tissue using ACE ImmunoID tumor with matched normal configuration where matched normal blood was available. Tumor mutational burden (TMB), assessed by whole-exome sequencing, was described according to the number of nonsynonymous somatic mutations (single-nucleotide variants and indels) per megabase.

### Pharmacokinetic Analyses

Systemic concentrations of avelumab and chemotherapies were measured in patient blood samples to estimate relevant pharmacokinetic parameters. Blood samples (3.5 mL) for avelumab pharmacokinetic analyses were collected prior to dosing and at the end of infusion on day 1 of cycles 1, 2, 3, 6, 10, and 14; additional samples were collected on day 15 of cycles 1, 2, and 3. Blood samples (3.5 mL) for avelumab immunogenicity analyses were collected prior to dosing on day 1 of cycles 1, 2, 3, 6, 10, and 14 and at the end of treatment.

### Statistical Analyses

Occurrence of DLT was assessed in the DLT analysis set and defined as all patients enrolled in the phase Ib who received ≥1 dose of combination treatment and either had DLT within the first two cycles of treatment or completed the DLT observation period. Patients who withdrew before receiving ≥75% of the planned dose of study treatment during the first two cycles for reasons other than treatment-related toxicity were not evaluable for DLT. Efficacy and safety were assessed in all patients who received ≥1 dose of study treatment. Pharmacokinetics was analyzed in all treated patients who had ≥1 postdose concentration measurement above the limit of quantitation. Observed maximum serum concentration (C_max_), trough serum concentration (C_trough_), and day 15 concentrations of avelumab at various cycles are reported. Immunogenicity was assessed in all patients with ≥1 antidrug antibody (ADA) sample. The Kaplan–Meier method was used to analyze DOR, PFS, and OS, and confidence intervals (CI) for median values were calculated using the Brookmeyer and Crowley method. CIs for ORRs were calculated using the Clopper–Pearson method.

### Data Availability Statement

Any requests for data by qualified scientific and medical researchers for legitimate research purposes will be subject to the healthcare business of Merck KGaA's (CrossRef Funder ID: 10.13039/100009945) Data Sharing Policy. All requests should be submitted in writing to the healthcare business of Merck KGaA's data sharing portal (https://www.emdgroup.com/en/research/our-approach-to-research-and-development/healthcare/clinical-trials/commitment-responsible-data-sharing.html). When the healthcare business of Merck KGaA has a co-research, co-development, or co-marketing or co-promotion agreement, or when the product has been out-licensed, the responsibility for disclosure might be dependent on the agreement between parties. Under these circumstances, the healthcare business of Merck KGaA will endeavor to gain agreement to share data in response to requests.

## Results

### Patients

Between January 15, 2018, and August 5, 2020, 66 patients were enrolled and started treatment at 24 centers, including 54 cisplatin-eligible patients with urothelial carcinoma and 12 patients with NSCLC. The data cutoff (last patient last visit) was December 20, 2022. In phase Ib, 19 patients with urothelial carcinoma received either avelumab 800 mg (*n* = 13) or 1,200 mg (*n* = 6) every 3 weeks in combination with cisplatin + gemcitabine, and 12 patients with NSCLC received either avelumab 800 mg (*n* = 6) or 1,200 mg (*n* = 6) every 3 weeks in combination with carboplatin + pemetrexed. Enrollment in the avelumab 1,200 mg cohort began approximately 9 months after the start of enrollment in the avelumab 800 mg cohort. In phase II, 35 patients with urothelial carcinoma received avelumab 1,200 mg every 3 weeks in combination with cisplatin + gemcitabine. Across all cohorts, most patients were White, male, and had an Eastern Cooperative Oncology Group performance status of 0, metastatic disease at enrollment, and no history of receiving neoadjuvant or adjuvant anticancer drug treatment (Supplementary Table S1). Most patients in the urothelial carcinoma cohorts had visceral disease. Tumors were PD-L1–positive (as determined by the Ventana PD-L1 SP263 IHC assay) in 34 patients (63.0%) with urothelial carcinoma and 1 patient (8.3%) with NSCLC.

In patients with urothelial carcinoma who received either avelumab dose, median (range) duration of treatment with avelumab, cisplatin, and gemcitabine was 28.6 (3.0–217.9), 15.2 (3.0–23.4), and 15.6 weeks (1.0–24.1), respectively (Supplementary Table S2). Median duration of treatment with avelumab 800 or 1,200 mg was 32.0 and 28.0 weeks, respectively. In patients with NSCLC who received either avelumab dose, median (range) duration of treatment with avelumab, carboplatin, and pemetrexed was 41.5 (9.9–216.6), 12.3 (9.9–21.0), and 26.5 weeks (9.9–210.0), respectively. Median duration of treatment with avelumab 800 or 1,200 mg was 55.1 and 21.7 weeks, respectively. The study was terminated at data cutoff, and patients who were still on treatment were enrolled in a continuation study.

### DLT

DLTs were assessed in patients enrolled in phase Ib. In DLT-evaluable patients with urothelial carcinoma, DLT occurred in 1 of 12 patients (8.3%) treated with avelumab 800 mg (grade 4 thrombocytopenia) and 1 of 6 patients (16.7%) treated with avelumab 1,200 mg (grade 2 asthenia, which resulted in failure to deliver ≥75% of the planned doses of study treatment within the first two cycles). No DLT was reported in the NSCLC cohorts. The recommended phase II dose for avelumab in combination with chemotherapy was 1,200 mg every 3 weeks in the urothelial carcinoma and NSCLC cohorts.

### Safety

Safety data, including all patients from phase Ib and phase II, are presented by tumor type and dose level in Table 1.

**TABLE 1:** Summary of safety in the urothelial carcinoma and NSCLC cohorts

	Urothelial carcinoma cohorts			NSCLC cohorts		
	Avelumab 800 mg + cisplatin + gemcitabine (*n* = 13)	Avelumab 1,200 mg + cisplatin + gemcitabine (*n* = 41)	Total urothelial carcinoma cohorts (*n* = 54)	Avelumab 800 mg + carboplatin + pemetrexed (*n* = 6)	Avelumab 1,200 mg + carboplatin + pemetrexed (*n* = 6)	Total NSCLC cohorts (*n* = 12)
Any-grade AE, *n* (%)	13 (100)	40 (97.6)	53 (98.1)	6 (100)	6 (100)	12 (100)
Grade ≥3	12 (92.3)	37 (90.2)	49 (90.7)	5 (83.3)	6 (100)	11 (91.7)
Any-grade TRAE, *n* (%)	12 (92.3)	40 (97.6)	52 (96.3)	6 (100)	6 (100)	12 (100)
Grade ≥3	11 (84.6)	34 (82.9)	45 (83.3)	4 (66.7)	4 (66.7)	8 (66.7)
AE leading to discontinuation of any study drug, *n* (%)	6 (46.2)	14 (34.1)	20 (37.0)	3 (50.0)	2 (33.3)	5 (41.7)
TRAE leading to discontinuation of any study drug, *n* (%)	3 (23.1)	13 (31.7)	16 (29.6)	3 (50.0)	2 (33.3)	5 (41.7)
AE leading to death, *n* (%)	—	2 (4.9)	2 (3.7)	—	1 (16.7)	1 (8.3)
TRAE leading to death, *n* (%)	—	—	—	—	1 (16.7)^a^	1 (8.3)
irAE, *n* (%)	5 (38.5)	9 (22.0)	14 (25.9)	4 (66.7)	2 (33.3)	6 (50.0)
Grade ≥3	2 (15.4)	4 (9.8)	6 (11.1)	3 (50.0)	2 (33.3)	5 (41.7)
irAE leading to death, *n* (%)	—	—	—	—	1 (16.7)	1 (8.3)
IRR, *n* (%)	—	6 (14.6)	6 (11.1)	1 (16.7)	—	1 (8.3)
Grade ≥3	—	2 (4.9)	2 (3.7)	—	—	—

^a^Pneumonitis.

### Urothelial Carcinoma

In total, 54 patients with urothelial carcinoma were treated with avelumab every 3 weeks + chemotherapy in phase Ib and phase II combined, including 13 patients treated with avelumab 800 mg and 41 patients treated with avelumab 1,200 mg. Treatment-related AEs (TRAE) of any grade and related to any study drug were reported in 52 of 54 patients (96.3%) with urothelial carcinoma, including grade 3 TRAEs in 45 patients (83.3%). Frequencies of TRAEs were similar in patients treated with avelumab 800 or 1,200 mg. No TRAEs leading to death were reported. The most common TRAEs of any grade in all patients with urothelial carcinoma were anemia (59.3%), neutropenia (55.6%), and nausea (50.0%), and the most common grade ≥3 TRAEs were neutropenia (38.9%), anemia (14.8%), and thrombocytopenia (14.8%; Table 2). TRAEs leading to discontinuation of any study drug occurred in 16 of 54 patients (29.6%), including 3 of 13 patients (23.1%) at the avelumab 800 mg dose level and 13 of 41 patients (31.7%) at the avelumab 1,200 mg dose level (Supplementary Table S3). Immune-related AEs (irAE) of any grade were reported in 14 of 54 patients (25.9%), including grade ≥3 irAEs in 6 patients (11.1%). At the avelumab 800 mg dose level, 5 of 13 patients (38.5%) had an irAE of any grade and 2 (15.4%) had a grade ≥3 irAE. At the avelumab 1,200 mg dose level, 9 of 41 patients (22.0%) had an irAE of any grade and 4 (9.8%) had a grade ≥3 irAE (Table 1). The most common irAEs of any grade by cluster were immune-related rash in 7 (13%) and immune-related hepatitis in 2 (3.7%), and the most common grade ≥3 irAE was immune-related hepatitis in 3 (3.7%; Supplementary Table S4). Infusion-related reactions (IRR) of any grade were reported in 6 of 54 patients (11.1%), including grade ≥3 IRRs in 2 patients (3.7%; Table 1).

**TABLE 2:** Summary of the most common TRAEs (any grade in ≥30% of patients or grade ≥3 in ≥10% of patients) in the urothelial carcinoma and NSCLC cohorts

	Urothelial cohorts			NSCLC cohorts		
	Avelumab 800 mg + cisplatin + gemcitabine (*n* = 13)	Avelumab 1,200 mg + cisplatin + gemcitabine (*n* = 41)	Total urothelial carcinoma cohorts (*n* = 54)	Avelumab 800 mg + carboplatin + pemetrexed (*n* = 6)	Avelumab 1,200 mg + carboplatin + pemetrexed (*n* = 6)	Total NSCLC cohorts (*n* = 12)
Any-grade TRAE, *n* (%)	12 (92.3)	40 (97.6)	52 (96.3)	6 (100)	6 (100)	12 (100)
Anemia	10 (76.9)	22 (53.7)	32 (59.3)	2 (33.3)	1 (16.7)	3 (25.0)
Neutropenia	8 (61.5)	22 (53.7)	30 (55.6)	3 (50.0)	4 (66.7)	7 (58.3)
Nausea	9 (69.2)	18 (43.9)	27 (50.0)	3 (50.0)	4 (66.7)	7 (58.3)
Thrombocytopenia	4 (30.8)	20 (48.8)	24 (44.4)	—	4 (66.7)	4 (33.3)
Fatigue	7 (53.8)	12 (29.3)	19 (35.2)	3 (50.0)	3 (50.0)	6 (50.0)
Platelet count decreased	4 (30.8)	9 (22.0)	13 (24.1)	3 (50.0)	1 (16.7)	4 (33.3)
Diarrhea	3 (23.1)	7 (17.1)	10 (18.5)	2 (33.3)	3 (50.0)	5 (41.7)
Grade ≥3 TRAE, *n* (%)	11 (84.6)	34 (82.9)	45 (83.3)	4 (66.7)	4 (66.7)	8 (66.7)
Neutropenia	7 (53.8)	14 (34.1)	21 (38.9)	2 (33.3)	3 (50.0)	5 (41.7)
Anemia	3 (23.1)	5 (12.2)	8 (14.8)	1 (16.7)	—	1 (8.3)
Thrombocytopenia	1 (7.7)	7 (17.1)	8 (14.8)	—	—	—
WBC count decreased	3 (23.1)	3 (7.3)	6 (11.1)	1 (16.7)	—	1 (8.3)
Neutrophil count decreased	3 (23.1)	3 (7.3)	6 (11.1)	—	—	—
Platelet count decreased	1 (7.7)	4 (9.8)	5 (9.3)	2 (33.3)	—	2 (16.7)
Pneumonitis	—	—	—	—	2 (33.3)	2 (16.7)

Abbreviations: NSCLC, non–small cell lung cancer; TRAE, treatment-related adverse event; UC, urothelial carcinoma; WBC, white blood cell.

### NSCLC

In total, 12 patients with NSCLC were treated with avelumab every 3 weeks + chemotherapy in phase Ib, including 6 patients each who received avelumab 800 or 1,200 mg. No patients with NSCLC were enrolled in phase II. TRAEs of any grade and related to any study drug were reported in all 12 patients with NSCLC, including grade ≥3 TRAEs in 8 patients (66.7%; Table 1). One patient with NSCLC died because of a TRAE of pneumonitis. The most common TRAEs of any grade in all patients with NSCLC were nausea (58.3%), neutropenia (58.3%), and fatigue (50.0%), and the most common grade ≥3 TRAEs were neutropenia (41.7%), platelet count decreased (16.7%), and pneumonitis (16.7%; Table 2). TRAEs leading to discontinuation of any study drug occurred in 5 of 12 patients (41.7%), including 3 of 6 patients (50%) at the avelumab 800 mg dose level and 2 of 6 patients (33.3%) at the avelumab 1,200 mg dose level (Supplementary Table S3). irAEs of any grade were reported in 6 of 12 patients (50%), including grade ≥3 irAEs in 5 patients (41.7%). At the avelumab 800 mg dose level, 4 of 6 patients (66.7%) had an irAE of any grade and 3 (50%) had grade ≥3 irAE. At the avelumab 1,200 mg dose level, 2 of 6 patients (33.3%) had an irAE of any grade and 2 (33.3%) had grade ≥3 irAEs (Table 1). The most common irAEs of any grade by clusters in all patients with NSCLC were immune-related pneumonitis (33.3%) and immune-related rash (25%), and the most common irAE grade ≥3 was immune-related pneumonitis 16.7%; Supplementary Table S4). IRRs of any grade were reported in 1 of 12 patients (8.3%); no grade ≥3 irAEs were reported in patients with NSCLC (Table 1).

### Antitumor Activity

Antitumor activity was assessed in all patients treated in the study, irrespective of study phase.

### Urothelial Carcinoma

**FIGURE 1:**
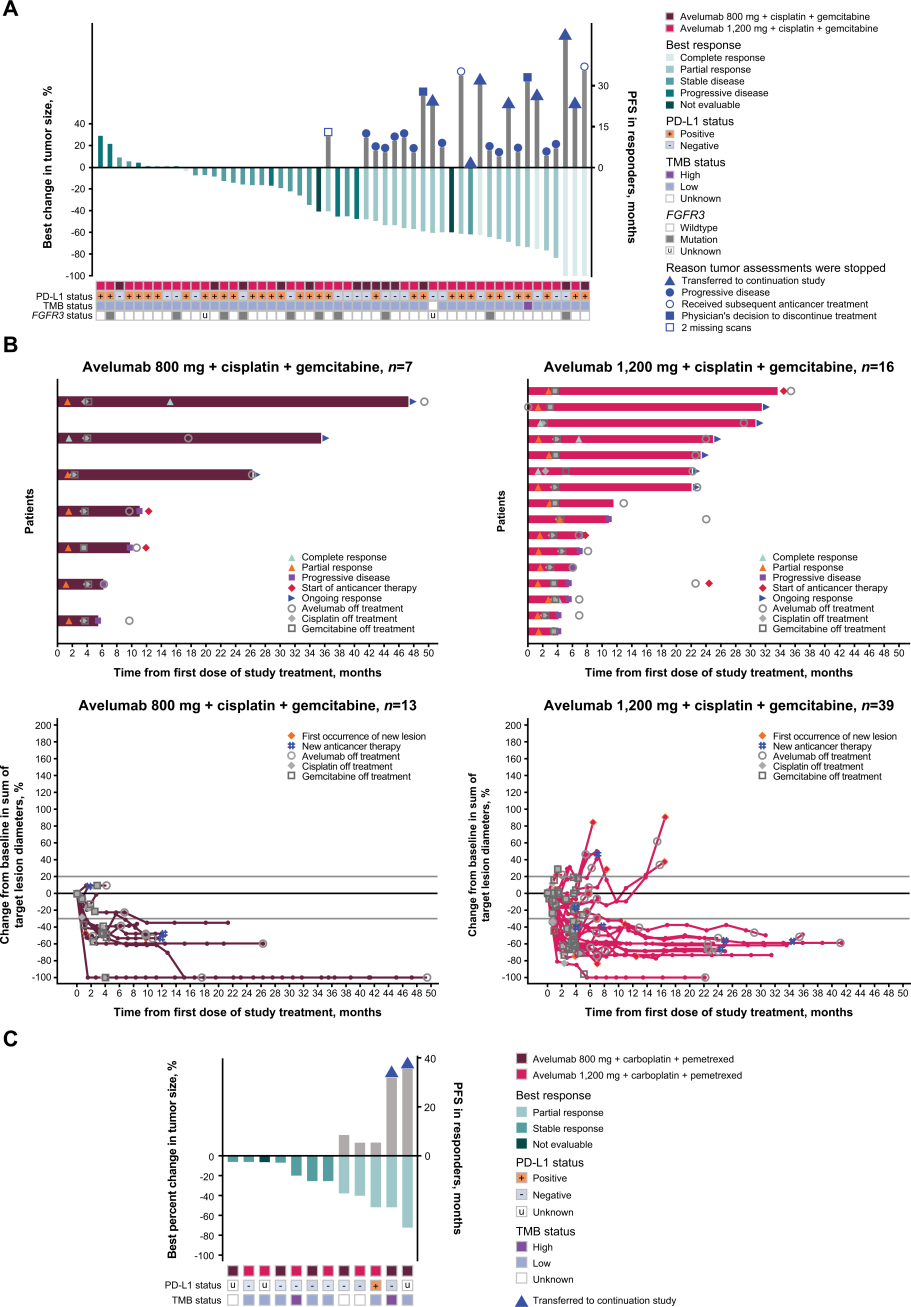
Summary of reductions in tumor size and durations of PFS or response. **A,** Best percentage change from baseline in target lesions (green bars) and PFS in responding patients (gray bars) in the urothelial carcinoma cohorts.^a^**B,** DOR in responding patients and change in tumor size over time in all patients in the urothelial carcinoma cohorts. **C,** Best percentage change from baseline in target lesions (green bars) and PFS in responding patients (gray bars) in the NSCLC cohorts. TMB high represents >10 mut/Mb. In urothelial carcinoma cohorts, PD-L1–positive status was defined using an algorithm that combines assessments of PD-L1 staining on tumor and immune cells, which were scored by pathologists (30); in NSCLC cohorts, PD-L1–positive status was defined as PD-L1 expression on ≥1% of tumor cells. PD-L1, programmed death-ligand 1; TMB, tumor mutational burden; FGFR, fibroblast growth factor receptor. ^a^Two patients with no postbaseline tumor assessments who were receiving avelumab 1,200 mg were excluded.

In the 54 patients with urothelial carcinoma who received either avelumab dose, the ORR was 42.6% [95% confidence interval (CI), 29.2–56.8], and in those treated with avelumab 800 mg (all enrolled in phase Ib) or 1,200 mg (enrolled in phase Ib or phase II), ORRs were 53.8% (95% CI, 25.1–80.8) and 39.0% (95% CI, 24.2–55.5), with CR rates of 15.4% and 7.3%, respectively (Table 3). Median TTR was 1.4 months (range, 1.1–4.3) and median DOR was 9.6 months [95% CI, 5.1–not evaluable (NE)], with responses ongoing in 7 patients at data cutoff. Long-term responses (lasting ≥15 months) were observed in 10 patients (Fig. 1). In all patients with urothelial carcinoma, median PFS was 5.5 months (95% CI, 3.1–7.0) and median OS was 15.1 months (95% CI, 9.4–22.2). In patients treated with avelumab 800 or 1,200 mg, median PFS (95% CI) was 9.8 months (2.2–NE) and 5.4 months (2.9–6.0), and median OS (95% CI) was 18.1 months (5.0–NE) and 15.1 months (8.7–22.0), respectively (Supplementary Fig. S1). Seven patients were alive and responding to treatment after 24 months of treatment (avelumab 800 mg, *n* = 4; avelumab 1,200 mg, *n* = 3), 2 patients (avelumab 800 mg) were alive and responding after 35 months, and 1 patient (avelumab 800 mg) was alive and responding after 45 months.

**TABLE 3:** Summary of efficacy in the urothelial carcinoma and NSCLC cohorts

	Urothelial carcinoma cohorts			NSCLC cohorts		
	Avelumab 800 mg + cisplatin + gemcitabine (*n* = 13)	Avelumab 1,200 mg + cisplatin + gemcitabine (*n* = 41)	Total urothelial carcinoma cohorts (*n* = 54)	Avelumab 800 mg + carboplatin + pemetrexed (*n* = 6)	Avelumab 1,200 mg + carboplatin + pemetrexed (*n* = 6)	Total NSCLC cohorts (*n* = 12)
Confirmed BOR, *n* (%)						
CR	2 (15.4)	3 (7.3)	5 (9.3)	—	—	—
PR	5 (38.5)	13 (31.7)	18 (33.3)	3 (50.0)	2 (33.3)	5 (41.7)
SD	5 (38.5)	13 (31.7)	18 (33.3)	3 (50.0)	3 (50.0)	6 (50.0)
PD	1 (7.7)	9 (22.0)	10 (18.5)	—	—	—
NE	—	3 (7.3)	3 (5.6)	—	1 (16.7)	1 (8.3)
ORR (95% CI), %	53.8 (25.1–80.8)	39.0 (24.2–55.5)	42.6 (29.2–56.8)	50.0 (11.8–88.2)	33.3 (4.3–77.7)	41.7 (15.2–72.3)
PFS, median (95% CI), months	9.8 (2.2-NE)	5.4 (2.9–6.0)	5.5 (3.1–7.0)	NA	NA	NA
OS, median (95% CI), months	18.1 (5.0–NE)	15.1 (8.7–22.0)	15.1 (9.4–22.0)	NA	NA	NA

Abbreviations: BOR, best overall response; NA, not assessed; PD, progressive disease; PR, partial response; SD, stable disease.

### NSCLC

In the 12 patients with NSCLC (all enrolled in phase Ib), the overall ORR was 41.7% (95% CI, 15.2–72.3), and in patients treated with avelumab 800 or 1,200 mg, ORRs (95% CI) were 50.0% (11.8–88.2) and 33.3% (4.3–77.7), respectively (Table 3). Two patients, who both received avelumab 800 mg, were still alive and responding to treatment after 45 months (Fig. 1). Median PFS and median OS were not derived because of the small number of patients.

### Biomarker Analyses

In the urothelial carcinoma cohort, ORRs (95% CIs) in patients treated with avelumab 800 mg who had PD-L1–positive (*n* = 6) or PD-L1–negative (*n* = 7) tumors were 50.0% (11.8–88.2) and 57.1% (18.4–90.1), respectively. ORRs (95% CI) in patients treated with avelumab 1,200 mg who had PD-L1–positive (*n* = 28) or PD-L1–negative (*n* = 13) tumors were 32.1% (15.9–52.4) and 53.8% (25.1–80.8), respectively (Supplementary Table S1). Best percentage changes from baseline in tumor size by PD-L1 and TMB status in patients with urothelial carcinoma and NSCLC are shown in Fig. 1. Of patients evaluable for TMB, 1 of 52 with urothelial carcinoma and 2 of 9 with NSCLC had high TMB (>10 mut/Mb), and associations with efficacy could not be assessed. Molecular profiling of evaluable urothelial carcinoma tumors showed that 10 of 52 (19.2%) patients had *FGFR3* mutations, with the most common mutation being S249C (*n* = 3; Fig. 1A). No associations between molecular profile and clinical outcome were observed.

### Pharmacokinetic and Immunogenicity Analyses

**FIGURE 2:**
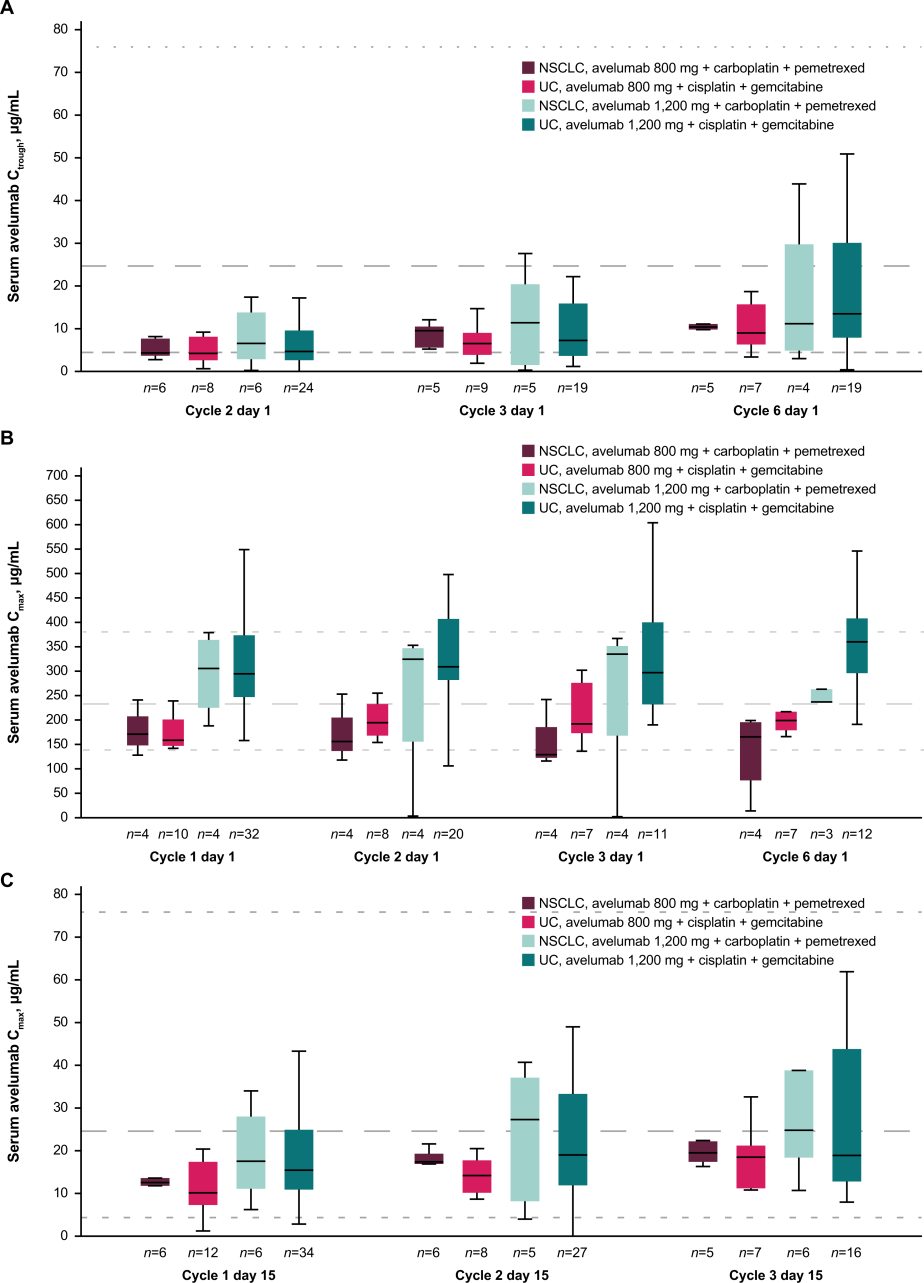
Pharmacokinetics of avelumab over time following administration of 800 and 1,200 mg every 3 weeks in the urothelial carcinoma and NSCLC cohorts. **A,** Serum avelumab C_trough_ by visit. **B,** Serum avelumab C_max_ by visit. **C,** Serum avelumab concentrations on day 15 visit. Horizontal black line within each box depicts the median, and upper and lower lines depict the third (Q3) and first (Q1) quantiles, respectively. Upper and lower error bars represent Q3 + 1.5 × IQR and Q1 – 1.5 × IQR, respectively. Dashed line represents median, and dotted lines represent 5th and 95th percentiles of historical steady state C_trough_ (A and C) or C_max_ (B) from population pharmacokinetic model–based simulations of avelumab 10 mg/kg every 2 weeks in monotherapy (1, 2). IQR, interquartile range.

Avelumab C_trough_, C_max_, and day 15 concentrations over time in all cohorts are shown in Fig. 2. Serum avelumab concentrations were similar at each dose in patients with urothelial carcinoma or NSCLC, with moderate to high variability observed; concentrations were generally higher with avelumab 1,200 versus 800 mg, most notably at C_max_.

The overall incidence of treatment-induced ADA response was 18.3%, ranging from 0% to 30% across the various treatment groups, with no evidence of higher ADA levels with higher avelumab dose. Quantitative differences in ADA response are not considered meaningful due to the relatively low incidence, small samples sizes, and difference in sample size between treatment groups.

## Discussion

The safety profile for avelumab in combination with cisplatin + gemcitabine in patients with urothelial carcinoma or in combination with carboplatin + pemetrexed in patients with NSCLC was similar to previous studies of other ICIs in combination with chemotherapy (14, 15, 33–37). No meaningful differences in baseline demographics or disease characteristics were observed between patients receiving avelumab 800 or 1,200 mg in the urothelial carcinoma or NSCLC cohorts. In the phase Ib safety lead-in cohorts, DLT occurred in 2 patients with urothelial carcinoma (grade 4 thrombocytopenia and grade 2 treatment-related asthenia at the avelumab 800-mg and 1,200-mg doses, respectively); no DLT was reported in patients with NSCLC. On the basis of these data, the recommended phase II dose for avelumab in combination with chemotherapy was 1,200 mg every 3 weeks in patients with urothelial carcinoma or NSCLC. Although the observed frequencies of DLT met the criteria to permit enrollment of both the urothelial carcinoma and NSCLC expansion cohorts in phase II, the study sponsor chose not to open the NSCLC expansion cohort because of changes in the treatment landscape since the study was initiated.

Across phase Ib and II, no new safety concerns were observed with any combination regimen. The safety profiles of avelumab and chemotherapies in this study were consistent with known toxicities of each individual drug, and the frequencies and severities of AEs with avelumab 800 mg every 2 weeks and 1,200 mg every 3 weeks were similar. In addition, despite the higher exposure confirmed by pharmacokinetic analyses, avelumab 1,200 mg every 3 weeks did not result in an increased risk of toxicity, suggesting that a higher dose administered every 3 weeks may be a tolerable alternative to 800 mg every 2 weeks.

Combination treatment with ICIs plus chemotherapy is standard of care in NSCLC but not urothelial carcinoma (5, 6). Efficacy results in this study were generally consistent with previous data for combination treatment with ICIs plus chemotherapy (14, 15, 35–38), although patient populations were small in this study. Long-term responses were observed in subsets of patients with urothelial carcinoma and NSCLC. Consistent with prior combination studies in urothelial carcinoma, the addition of avelumab to platinum-based chemotherapy did not result in higher response rates compared with platinum-based chemotherapy in previous trials (42.6% vs. 44%-49%, respectively; refs. 14, 16, 38). On the basis of results from the phase III JAVELIN Bladder 100 trial, avelumab administered as 1 L maintenance treatment is standard of care in patients with advanced urothelial carcinoma that has not progressed with 1 L platinum-based chemotherapy (1, 2, 6, 17). In patients with NSCLC, the ORR with avelumab plus platinum-based chemotherapy was similar or lower than ORRs observed in other ICI-based combination studies in patients with nonsquamous NSCLC (41.7% vs. 43%–55%, respectively; refs. 33, 35–37).

Avelumab pharmacokinetic exposures at 800 mg every 3 weeks or 1,200 mg every 3 weeks were as predicted and overlapped with previous model-based simulations of the approved 800 mg every 2 weeks regimen administered as monotherapy (historical control; ref. 25), with no evidence of meaningful changes in avelumab exposure to suggest a drug interaction. The overall incidence of immunogenicity in this study is comparable to that in earlier studies of avelumab (1).

In biomarker analyses, no association was detected between tumor PD-L1 status and antitumor activity. Fewer than expected patients had high TMB (≥10 mut/Mb), particularly in the urothelial carcinoma cohort, and the median TMB (1.68 mut/Mb) was lower than that reported in the phase III JAVELIN Bladder 100 trial, which may indicate lower tumor immunogenicity (39). Biomarker analyses were limited by the small patient numbers within the cohorts, hence interpretation of results was limited.

This study was not designed to formally compare safety, efficacy, or pharmacokinetics between avelumab 800 and 1,200 mg, and further conclusions cannot be drawn due to the limited number of patients. The therapeutic landscapes in advanced urothelial carcinoma and NSCLC have evolved since the initiation of this trial, and the combination of an antibody–drug conjugate with an ICI has recently demonstrated improved clinical activity compared with platinum-based chemotherapy in patients with advanced urothelial carcinoma (40). Consequently, no further evaluation of avelumab + chemotherapy combinations is planned in these tumor types.

## Conclusions

Treatment with avelumab 1,200 mg every 3 weeks in combination with cisplatin + gemcitabine in patients with advanced urothelial carcinoma and in combination with carboplatin + pemetrexed in patients with advanced nonsquamous NSCLC is feasible. Avelumab pharmacokinetic exposures at 800 mg every 3 weeks or 1,200 mg every 3 weeks overlapped with those of the approved 800 mg every 2 weeks regimen. Thus, a higher dose of avelumab with less frequent administration may be a tolerable alternative treatment regimen. However, results from this study, and changes in the treatment landscape since the study was initiated, do not support further studies of avelumab in combination with platinum-based chemotherapy in these tumor types.

## Supplementary Material

Supplementary Data Supplementary Figure 1

Supplementary Data Supplementary Table 1

Supplementary Data Supplementary Table 2

Supplementary Data Supplementary Table 3

Supplementary Data Supplementary Table 4

## References

[1] Bavencio (avelumab). Prescribing information. EMD Serono, Rockland, MA, USA; 2023.

[2] Bavencio (avelumab). Summary of product characteristics. Merck Europe B.V., Amsterdam, Netherlands, an affiliate of Merck KGaA, Darmstadt, Germany; 2023.

[3] Verschraegen CF , Jerusalem G, McClay EF, Iannotti N, Redfern CH, Bennouna J, . Efficacy and safety of first-line avelumab in patients with advanced non-small cell lung cancer: results from a phase Ib cohort of the JAVELIN Solid Tumor study. J Immunother Cancer 2020;8:e001064. 32907924 10.1136/jitc-2020-001064 PMC7481079

[4] Reck M , Bariesi F, Yang JC-H, Westeel V, Felip E, Özgüroğlu M, . Avelumab vs chemotherapy for first-line treatment of advanced PD-L1+ NSCLC: primary analysis from JAVELIN Lung 100. J Thorac Oncol 2022;17:9. 10.1016/j.jtho.2023.09.1445 37748693

[5] NCCN Clinical Practice Guidelines in Oncology. Non-small cell lung cancer. V3; 2023.

[6] NCCN Clinical Practice Guidelines in Oncology. Bladder cancer. V3; 2023.

[7] de Biasi AR , Villena-Vargas J, Adusumilli PS. Cisplatin-induced antitumor immunomodulation: a review of preclinical and clinical evidence. Clin Cancer Res 2014;20:5384–91. 25204552 10.1158/1078-0432.CCR-14-1298 PMC4216745

[8] Zheng H , Zeltsman M, Zauderer MG, Eguchi T, Vaghjiani RG, Adusumilli PS. Chemotherapy-induced immunomodulation in non-small-cell lung cancer: a rationale for combination chemoimmunotherapy. Immunotherapy 2017;9:913–27. 29338609 10.2217/imt-2017-0052 PMC5810850

[9] Heinhuis KM , Ros W, Kok M, Steeghs N, Beijnen JH, Schellens JHM. Enhancing antitumor response by combining immune checkpoint inhibitors with chemotherapy in solid tumors. Ann Oncol 2019;30:219–35. 30608567 10.1093/annonc/mdy551

[10] Zitvogel L , Galluzzi L, Smyth MJ, Kroemer G. Mechanism of action of conventional and targeted anticancer therapies: reinstating immunosurveillance. Immunity 2013;39:74–88. 23890065 10.1016/j.immuni.2013.06.014

[11] Keytruda (pembrolizumab). Prescribing information. Merck & Co., Kenilworth, NJ, USA; 2022.

[12] Opdivo (nivolumab). Prescribing information. Bristol Myers Squibb Company; 2022.

[13] Tecentriq (atezolizumab). Prescribing information. Genentech; 2021.

[14] Powles T , Csőszi T, Özgüroğlu M, Matsubara N, Géczi L, Cheng SY, . Pembrolizumab alone or combined with chemotherapy versus chemotherapy as first-line therapy for advanced urothelial carcinoma (KEYNOTE-361): a randomised, open-label, phase 3 trial. Lancet Oncol 2021;22:931–45. 34051178 10.1016/S1470-2045(21)00152-2

[15] Galsky MD , Arija JÁA, Bamias A, Davis ID, De Santis M, Kikuchi E, . Atezolizumab with or without chemotherapy in metastatic urothelial cancer (IMvigor130): a multicentre, randomised, placebo-controlled phase 3 trial. Lancet 2020;395:1547–57. 32416780 10.1016/S0140-6736(20)30230-0

[16] Bamias A , Davis ID, Galsky MD, Arija JÁA, Kikuchi E, Grande E, . Final overall survival (OS) analysisof atezolizumab (atezo) monotherapy vs chemotherapy (chemo) in untreated locally advanced or metastatic urothelial carcinoma (mUC) from the Phase 3 IMvigor130 study. J Clin Oncol 41: 6s, 2023 (suppl; abstr LBA441).

[17] Powles T , Park SH, Voog E, Caserta C, Valderrama BP, Gurney H, . Avelumab maintenance therapy for advanced or metastatic urothelial carcinoma. N Engl J Med 2020;383:1218–30. 32945632 10.1056/NEJMoa2002788

[18] Powles T , Park SH, Caserta C, Valderrama BP, Gurney H, Ullén A, . Avelumab first-line maintenance for advanced urothelial carcinoma: results from the JAVELIN Bladder 100 trial after ≥2 years of follow-up. J Clin Oncol 2023;41:3486–92. 37071838 10.1200/JCO.22.01792 PMC10306435

[19] Powles T , Bellmunt J, Comperat E, De Santis M, Huddart R, Loriot Y, . Bladder cancer: ESMO clinical practice guideline for diagnosis, treatment and follow-up. Ann Oncol 2022;33:244–58. 34861372 10.1016/j.annonc.2021.11.012

[20] Witjes JA , Bruins HM, Cathomas R, Compérat EM, Cowan NC, Gakis G, . European association of urology guidelines on muscle-invasive and metastatic bladder cancer: summary of the 2020 guidelines. Eur Urol 2021;79:82–104. 32360052 10.1016/j.eururo.2020.03.055

[21] Motzer RJ , Penkov K, Haanen J, Rini B, Albiges L, Campbell MT, . Avelumab plus axitinib versus sunitinib for advanced renal-cell carcinoma. N Engl J Med 2019;380:1103–15. 30779531 10.1056/NEJMoa1816047 PMC6716603

[22] Apolo AB , Ellerton JA, Infante JR, Agrawal M, Gordon MS, Aljumaily R, . Avelumab as second-line therapy for metastatic, platinum-treated urothelial carcinoma in the phase Ib JAVELIN Solid Tumor study: 2-year updated efficacy and safety analysis. J Immunother Cancer 2020;8:e001246. 33037118 10.1136/jitc-2020-001246 PMC7549450

[23] D'Angelo SP , Bhatia S, Brohl AS, Hamid O, Mehnert JM, Terheyden P, . Avelumab in patients with previously treated metastatic Merkel cell carcinoma: long-term data and biomarker analyses from the single-arm phase 2 JAVELIN Merkel 200 trial. J Immunother Cancer 2020;8:e000674. 32414862 10.1136/jitc-2020-000674 PMC7239697

[24] D'Angelo SP , Lebbé C, Mortier L, Brohl AS, Fazio N, Grob JJ, . First-line avelumab in a cohort of 116 patients with metastatic Merkel cell carcinoma (JAVELIN Merkel 200): primary and biomarker analyses of a phase II study. J Immunother Cancer 2021;9:e002646. 34301810 10.1136/jitc-2021-002646 PMC8311489

[25] Novakovic AM , Wilkins JJ, Dai H, Wade JR, Neuteboom B, Brar S, . Changing body weight-based dosing to a flat dose for avelumab in metastatic merkel cell and advanced urothelial carcinoma. Clin Pharmacol Ther 2020;107:588–96. 31553054 10.1002/cpt.1645 PMC7027979

[26] Heery CR , O'Sullivan-Coyne G, Madan RA, Cordes L, Rajan A, Rauckhorst M, . Avelumab for metastatic or locally advanced previously treated solid tumours (JAVELIN Solid Tumor): a phase 1a, multicohort, dose-escalation trial. Lancet Oncol 2017;18:587–98. 28373007 10.1016/S1470-2045(17)30239-5 PMC6387686

[27] Herrera AF , Burton C, Radford J, Miall F, Townsend W, Santoro A, . Avelumab in relapsed/refractory classical Hodgkin lymphoma: phase 1b results from the JAVELIN Hodgkins trial. Blood Adv 2021;5:3387–96. 34477818 10.1182/bloodadvances.2021004511 PMC8525219

[28] Platinol (cisplatin). Prescribing information. Bristol-Myers Squibb; 2019.

[29] Gemzar (gemcitabine). Prescribing information. Eli Lilly; 2019.

[30] Alimta (pemetrexed disodium). Prescribing information. Eagle Pharmaceuticals, Inc; 2022.

[31] Paraplatin (carboplatin). Prescribing information. Pfizer; 2022.

[32] Zajac M , Boothman AM, Ben Y, Gupta A, Jin X, Mistry A, . Analytical validation and clinical utility of an immunohistochemical programmed death ligand-1 diagnostic assay and combined tumor and immune cell scoring algorithm for durvalumab in urothelial carcinoma. Arch Pathol Lab Med 2019;143:722–31. 30457897 10.5858/arpa.2017-0555-OA

[33] Langer CJ , Gadgeel SM, Borghaei H, Papadimitrakopoulou VA, Patnaik A, Powell SF, . Carboplatin and pemetrexed with or without pembrolizumab for advanced, non-squamous non-small-cell lung cancer: a randomised, phase 2 cohort of the open-label KEYNOTE-021 study. Lancet Oncol 2016;17:1497–508. 27745820 10.1016/S1470-2045(16)30498-3 PMC6886237

[34] Paz-Ares L , Luft A, Vicente D, Tafreshi A, Gümüş M, Mazières J, . Pembrolizumab plus chemotherapy for squamous non-small-cell lung cancer. N Engl J Med 2018;379:2040–51. 30280635 10.1056/NEJMoa1810865

[35] Gandhi L , Rodriguez-Abreu D, Gadgeel S, Esteban E, Felip E, De Angelis F, . Pembrolizumab plus chemotherapy in metastatic non-small-cell lung cancer. N Engl J Med 2018;378:2078–92. 29658856 10.1056/NEJMoa1801005

[36] Nishio M , Barlesi F, West H, Ball S, Bordoni R, Cobo M, . Atezolizumab plus chemotherapy for first-line treatment of nonsquamous NSCLC: results from the randomized phase 3 IMpower132 trial. J Thorac Oncol 2021;16:653–64. 33333328 10.1016/j.jtho.2020.11.025

[37] Gogishvili M , Melkadze T, Makharadze T, Giorgadze D, Dvorkin M, Penkov K, . Cemiplimab plus chemotherapy versus chemotherapy alone in non-small cell lung cancer: a randomized, controlled, double-blind phase 3 trial. Nat Med 2022;28:2374–80. 36008722 10.1038/s41591-022-01977-y PMC9671806

[38] Powles T , van der Heijden MS, Castellano D, Galsky MD, Loriot Y, Petrylak DP, . Durvalumab alone and durvalumab plus tremelimumab versus chemotherapy in previously untreated patients with unresectable, locally advanced or metastatic urothelial carcinoma (DANUBE): a randomised, open-label, multicentre, phase 3 trial. Lancet Oncol 2020;21:1574–88. 32971005 10.1016/S1470-2045(20)30541-6

[39] Powles T , Sridhar SS, Loriot Y, Bellmunt J, Mu XJ, Ching KA, . Avelumab maintenance in advanced urothelial carcinoma: biomarker analysis of the phase 3 JAVELIN Bladder 100 trial. Nat Med 2021;27:2200–11. 34893775 10.1038/s41591-021-01579-0

[40] Powles TB , Valderrama BP, Gupta S, Bedke J, Kikuchi E, Hoffman-Censits J, . LBA6 EV-302/KEYNOTE-A39: open-label, randomized phase III study of enfortumab vedotin in combination with pembrolizumab (EV+P) vs chemotherapy (Chemo) in previously untreated locally advanced metastatic urothelial carcinoma (la/mUC). Ann Oncol 2023;34:S1340.

