# Supplementary material for: First-line Avelumab plus Chemotherapy in Patients with Advanced Solid Tumors: Results from the Phase Ib/II JAVELIN Chemotherapy Medley Study
Source: Cancer Res Commun. 2024 Jun 28;4(6):1609–19. doi: 10.1158/2767-9764.CRC-23-0459 (PMC11212597; doi:10.1158/2767-9764.CRC-23-0459)
Supplement: Supplementary Data — Supplementary Figure 1 [file crc-23-0459-s01.docx]

**Supplementary Figure S1.** Kaplan-Meier analysis of (A) PFS and (B) OS in the urothelial carcinoma cohorts.


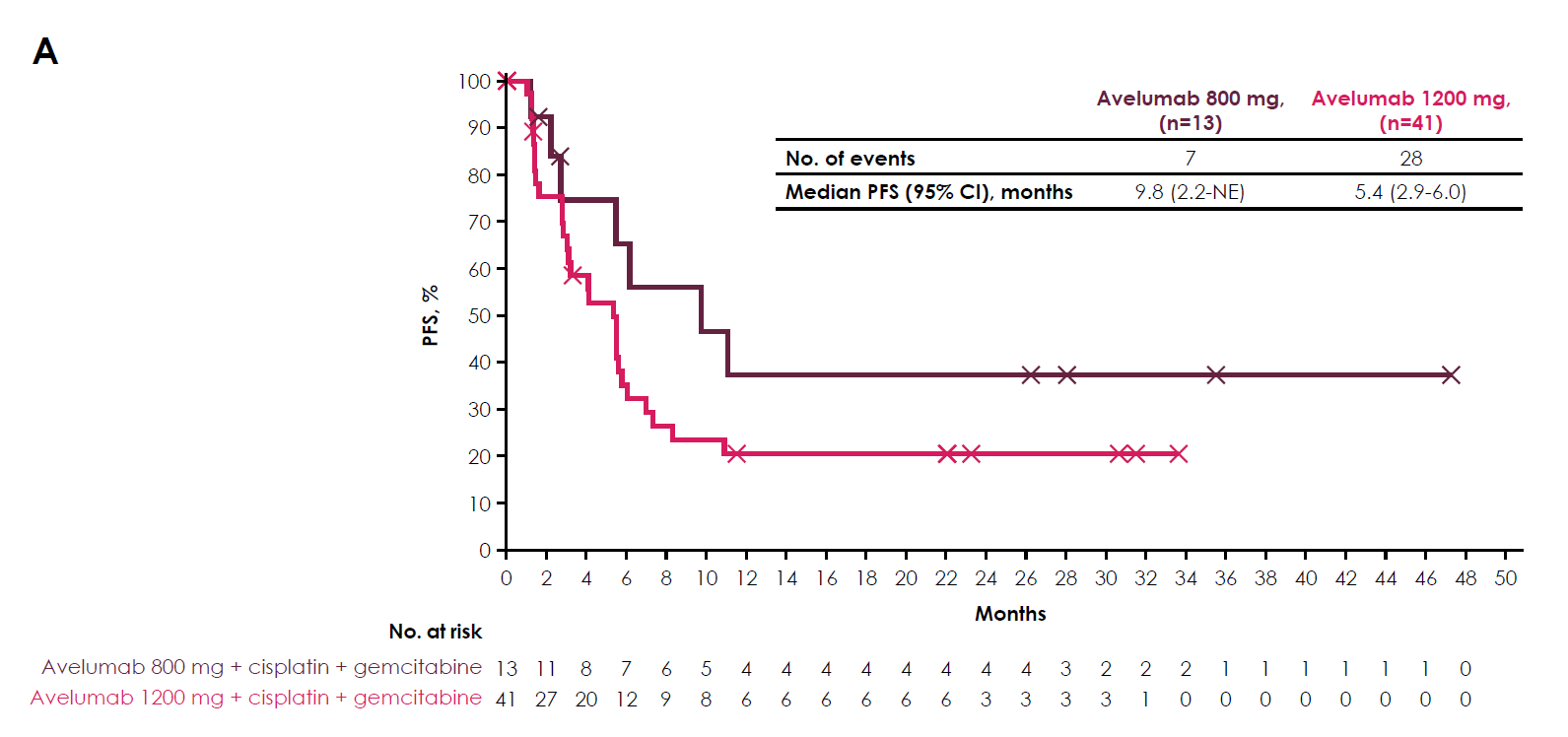


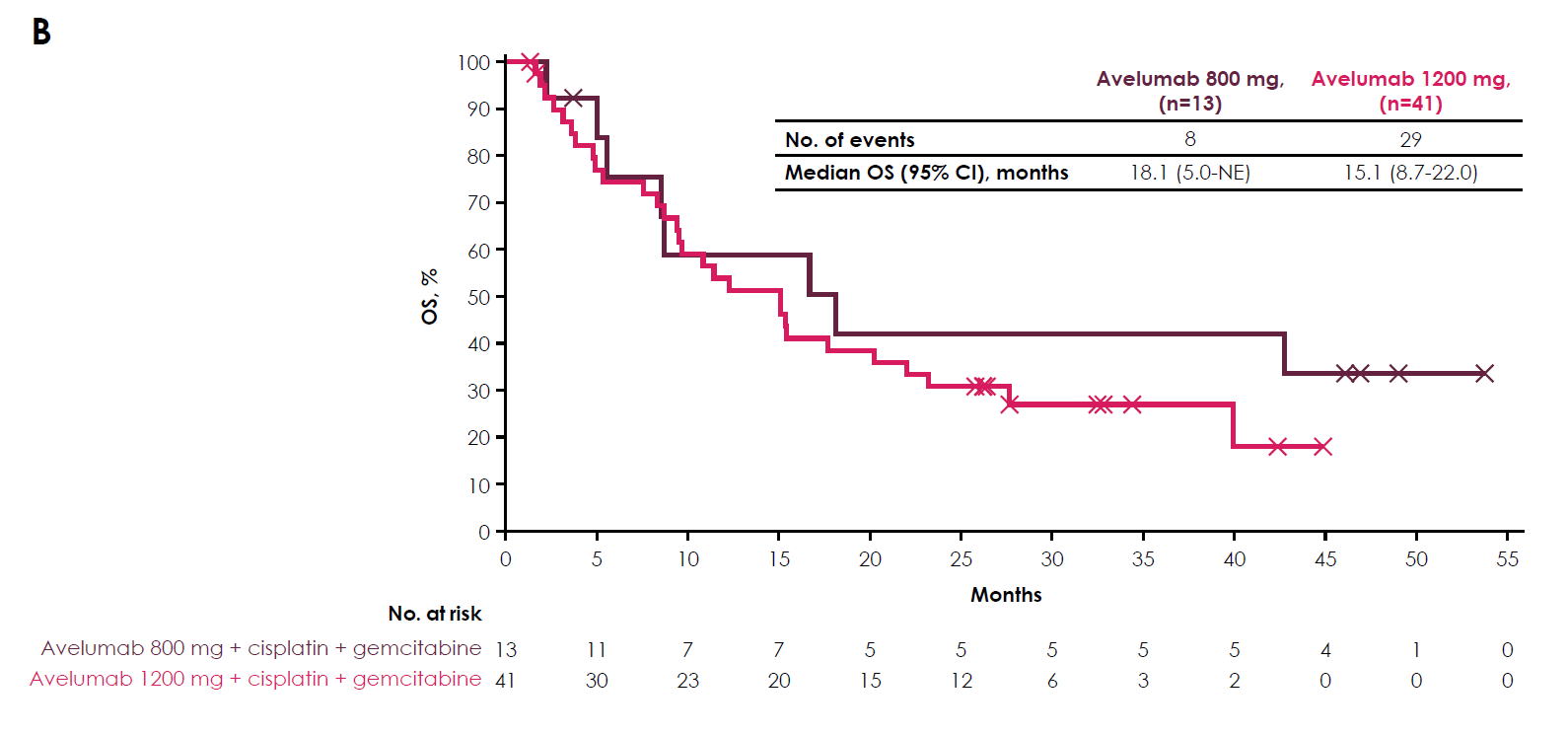


**NE**, not estimable; **OS**, overall survival; **PFS**, progression-free survival; **UC**, urothelial carcinoma
