# Supplementary material for: First-line Avelumab plus Chemotherapy in Patients with Advanced Solid Tumors: Results from the Phase Ib/II JAVELIN Chemotherapy Medley Study
Source: Cancer Res Commun. 2024 Jun 28;4(6):1609–19. doi: 10.1158/2767-9764.CRC-23-0459 (PMC11212597; doi:10.1158/2767-9764.CRC-23-0459)
Supplement: Supplementary Data — Supplementary Table 1 [file crc-23-0459-s02.docx]

**Supplementary Table S1.** Patient baseline characteristics and demographics in the urothelial carcinoma and NSCLC cohorts.

|  | **Urothelial carcinoma cohorts** | | | **NSCLC cohorts** | | |
| --- | --- | --- | --- | --- | --- | --- |
|  | **Avelumab 800 mg + cisplatin + gemcitabine (n=13)** | **Avelumab 1200 mg + cisplatin + gemcitabine (n=41)** | **Total urothelial carcinoma cohorts  (N=54)** | **Avelumab 800 mg + carboplatin + pemetrexed (n=6)** | **Avelumab 1200 mg + carboplatin + pemetrexed (n=6)** | **Total NSCLC cohorts (N=12)** |
| **Age, median (range), years** | 64.0 (42.0-76.0) | 67.0 (38.0-81.0) | 67.0 (38.0-81.0) | 69.0 (61.0-79.0) | 65.5 (54.0-78.0) | 67.0 (54.0-79.0) |
| **Sex, n (%)**  **Male**  **Female** | 8 (61.5)  5 (38.5) | 31 (75.6)  10 (24.4) | 39 (72.2)  15 (27.8) | 4 (66.7)  2 (33.3) | 6 (100)  – | 10 (83.3)  2 (16.7) |
| **Race, n (%)**  **White**  **Black or African American**  **Asian**  **Not reported** | 11 (84.6)  1 (7.7)  1 (7.7)  – | 37 (90.2)  –  1 (2.4)  3 (7.3) | 48 (88.9)  1 (1.9)  2 (3.7)  3 (5.6) | 6 (100.0)  –  –  – | 5 (83.3)  –  1 (16.7)  – | 11 (91.7)  –  1 (8.3)  – |
| **Geographic region, n (%)**  **North America**  **Europe**  **Asia**  **Rest of the world** | 3 (23.1)  9 (69.2)  –  1 (7.7) | 1 (2.4)  38 (92.7)  –  2 (4.9) | 4 (7.4)  47 (87.0)  –  3 (5.6) | 2 (33.3)  4 (66.7)  –  – | –  3 (50.0)  –  3 (50.0) | 2 (16.7)  7 (58.3)  –  3 (25.0) |
| **ECOG PS, n (%)**  **0**  **1** | 8 (61.5)  5 (38.5) | 26 (63.4)  15 (36.6) | 34 (63.0)  20 (37.0) | 5 (83.3)  1 (16.7) | 3 (50.0)  3 (50.0) | 8 (66.7)  4 (33.3) |
| **PD-L1 status, n (%)^a^**  **Positive**  **Negative**  **Unknown** | 6 (46.2)  7 (53.8)  – | 28 (68.3)  13 (31.7)  – | 34 (63.0)  20 (37.0)  – | –  4 (66.7)  2 (33.3) | 1 (16.7)  4 (66.7)  1 (16.7) | 1 (8.3)  8 (66.7)  3 (25.0) |
| **Visceral disease, n (%)**  **Yes**  **No** | 11 (84.6)  2 (15.4) | 34 (82.9)  7 (17.1) | 45 (83.3)  9 (16.7) | – | – | – |
| **TNM stage at diagnosis, n (%)**  **III**  **IV** | 3 (23.1)  7 (53.8) | 5 (12.2)  18 (43.9) | 8 (14.8)  25 (46.3) | –  6 (100.0) | 1 (16.7)  5 (83.3) | 1 (8.3)  11 (91.7) |
| **Prior anticancer drug regimens, n (%)**^b^  **0**  **1**  **2** | 11 (84.6)  1 (7.7)  1 (7.7) | 29 (70.7)  10 (24.4)  2 (4.9) | 40 (74.1)  11 (20.4)  3 (5.6) | 6 (100.0)  –  – | 5 (83.3)  1 (16.7)  – | 11 (91.7)  1 (8.3)  – |

**ECOG PS**, Eastern Cooperative Oncology Group performance status.

^a^In urothelial carcinoma cohorts, PD-L1–positive status was defined using an algorithm that combines assessments of PD-L1 staining on tumor and immune cells, which were scored by pathologists^30^; in NSCLC cohorts, PD-L1–positive status was defined as PD-L1 expression on ≥1% of tumor cells.

^b^Prior systemic chemotherapy in the adjuvant or neoadjuvant setting was permitted in patients with NSCLC who had a disease-free interval of ≥6 months and in patients with urothelial carcinoma who had a disease-free interval of ≥12 months.
