# Supplementary material for: First-line Avelumab plus Chemotherapy in Patients with Advanced Solid Tumors: Results from the Phase Ib/II JAVELIN Chemotherapy Medley Study
Source: Cancer Res Commun. 2024 Jun 28;4(6):1609–19. doi: 10.1158/2767-9764.CRC-23-0459 (PMC11212597; doi:10.1158/2767-9764.CRC-23-0459)
Supplement: Supplementary Data — Supplementary Table 2 [file crc-23-0459-s03.docx]

**Supplementary Table S2.** Duration of therapy in the urothelial carcinoma and NSCLC cohorts.

|  | **Urothelial carcinoma cohorts** | | | **NSCLC cohorts** | | |
| --- | --- | --- | --- | --- | --- | --- |
|  | **Avelumab 800 mg + cisplatin + gemcitabine (n=13)** | **Avelumab 1200 mg + cisplatin + gemcitabine (n=41)** | **Total urothelial carcinoma cohorts  (N=54)** | **Avelumab 800 mg + carboplatin + pemetrexed (n=6)** | **Avelumab 1200 mg + carboplatin + pemetrexed (n=6)** | **Total NSCLC cohorts  (N=12)** |
| **Duration of avelumab therapy, median (range), weeks** | 32.0 (3.0-217.9) | 28.0 (3.0-182.0) | 28.6 (3.0-217.9) | 55.1 (10.1-216.6) | 21.7 (9.9-66.3) | 41.5 (9.9-216.6) |
| **Duration of cisplatin therapy, median (range), weeks** | 15.3 (3.0-20.0) | 15.1 (3.0-23.4) | 15.2 (3.0-23.4) | – | – | – |
| **Duration of gemcitabine therapy, median (range), weeks** | 13.3 (1.0-20.0) | 16.0 (1.0-24.1) | 15.6 (1.0-24.1) | – | – | – |
| **Duration of carboplatin therapy, median (range), weeks** | – | – | – | 15.1 (10.1-21.0) | 12.3 (9.9-19.4) | 12.3 (9.9-21.0) |
| **Duration of pemetrexed therapy, median (range), weeks** | – | – | – | 33.5 (10.1-210.0) | 21.7 (9.9-66.3) | 26.5 (9.9-210.0) |

**NSCLC**, non-small cell lung cancer; **UC**, urothelial carcinoma.
