# Supplementary material for: First-line Avelumab plus Chemotherapy in Patients with Advanced Solid Tumors: Results from the Phase Ib/II JAVELIN Chemotherapy Medley Study
Source: Cancer Res Commun. 2024 Jun 28;4(6):1609–19. doi: 10.1158/2767-9764.CRC-23-0459 (PMC11212597; doi:10.1158/2767-9764.CRC-23-0459)
Supplement: Supplementary Data — Supplementary Table 3 [file crc-23-0459-s04.docx]

**Supplementary Table S3.** TRAEs leading to discontinuation of any study drug in >1 patient in the urothelial carcinoma or NSCLC cohorts.

|  | **Urothelial carcinoma cohorts** | | | **NSCLC cohorts** | | |
| --- | --- | --- | --- | --- | --- | --- |
|  | **Avelumab 800 mg + cisplatin + gemcitabine (n=13)** | **Avelumab 1200 mg + cisplatin + gemcitabine (n=41)** | **Total urothelial cohorts  (N=54)** | **Avelumab 800 mg + carboplatin + pemetrexed (n=6)** | **Avelumab 1200 mg + carboplatin + pemetrexed (n=6)** | **Total NSCLC cohorts  (N=12)** |
| **AE leading to discontinuation of any study drug, n (%)** | 3 (23.1) | 13 (31.7) | 16 (29.6) | 3 (50.0) | 2 (33.3) | 5 (41.7) |
| Neutropenia | 2 (15.4) | 2 (4.9) | 4 (7.4) | – | – | – |
| Thrombocytopenia | – | 3 (7.3) | 3 (5.6) | – | – | – |
| Anemia | – | 2 (4.9) | 2 (3.7) | – | – | – |
| Blood creatinine increased | – | – | – | 2 (33.3) | – | 2 (16.7) |
| Pneumonitis | – | – | – | – | 2 (33.3) | 2 (16.7) |

**AE**, adverse event; **NSCLC**, non-small cell lung cancer; **TRAE**, treatment-related adverse event; **UC**, urothelial carcinoma.
