# Supplementary material for: First-line Avelumab plus Chemotherapy in Patients with Advanced Solid Tumors: Results from the Phase Ib/II JAVELIN Chemotherapy Medley Study
Source: Cancer Res Commun. 2024 Jun 28;4(6):1609–19. doi: 10.1158/2767-9764.CRC-23-0459 (PMC11212597; doi:10.1158/2767-9764.CRC-23-0459)
Supplement: Supplementary Data — Supplementary Table 4 [file crc-23-0459-s05.docx]

**Supplementary Table S4.** Summary of irAEs in the urothelial carcinoma and NSCLC cohorts.

| **irAE clusters** | **Urothelial carcinoma cohorts** | | | **NSCLC cohorts** | | |
| --- | --- | --- | --- | --- | --- | --- |
|  | **Avelumab 800 mg + cisplatin + gemcitabine (n=13)** | **Avelumab 1200 mg + cisplatin + gemcitabine (n=41)** | **Total urothelial carcinoma cohorts  (N=54)** | **Avelumab 800 mg + carboplatin + pemetrexed (n=6)** | **Avelumab 1200 mg + carboplatin + pemetrexed (n=6)** | **Total NSCLC cohorts  (N=12)** |
| **Any-grade irAE, n (%)** | **5 (38.5)** | **9 (22.0)** | **14 (25.9)** | **4 (66.7)** | **2 (33.3)** | **6 (50.0)** |
| Immune-related rash | 3 (23.1) | 4 (9.8) | 7 (13.0) | 2 (33.3) | 1 (16.7) | 3 (25.0) |
| Immune-related hepatitis | 1 (7.7) | 1 (2.4) | 2 (3.7) | 1 (16.7) | – | 1 (8.3) |
| Immune-related colitis | – | 1 (2.4) | 1 (1.9) | 1 (16.7) | – | 1 (8.3) |
| Immune-related adrenal insufficiency | – | 1 (2.4) | 1 (1.9) | – | – | – |
| Immune-related thyroid disorders | – | 1 (2.4) | 1 (1.9) | 1 (16.7) | – | 1 (8.3) |
| Immune-related nephritis/renal dysfunction | – | 1 (2.4) | 1 (1.9) | 1 (16.7) | – | 1 (8.3) |
| Immune-related pancreatitis | 1 (7.7) | – | 1 (1.9) | – | – | – |
| Immune-related myositis | – | 1 (2.4) | 1 (1.9) | – | – | – |
| Psoriasis | 1 (7.7) | – | 1 (1.9) | – | – | – |
| Immune-related pneumonitis | – | – | – | 2 (33.3) | 2 (33.3) | 4 (33.3) |
| Immune-related pituitary dysfunction | – | – | – | 1 (16.7) | – | 1 (8.3) |
| **Grade ≥3 irAE, n (%)** | **2 (15.4)** | **4 (9.8)** | **6 (11.1)** | **3 (50.0)** | **2 (33.3)** | **5 (41.7)** |
| Immune-related hepatitis | 1 (7.7) | 1 (2.4) | 2 (3.7) | 1 (16.7) | – | 1 (8.3) |
| Immune-related rash | 1 (7.7) | – | 1 (1.9) | – | – | – |
| Immune-related colitis | – | 1 (2.4) | 1 (1.9) | 1 (16.7) | – | 1 (8.3) |
| Immune-related nephritis/renal dysfunction | – | 1 (2.4) | 1 (1.9) | 1 (16.7) | – | 1 (8.3) |
| Immune-related pancreatitis | 1 (7.7) | – | 1 (1.9) | – | – | – |
| Immune-related myositis | – | 1 (2.4) | 1 (1.9) | – | – | – |
| Immune-related pneumonitis | – | – | – | – | 2 (33.3) | 2 (16.7) |

**irAE**, immune-related adverse event; **NSCLC**, non-small cell lung cancer; **UC**, urothelial carcinoma.
